# Exploring mental well-being, the emotional impact of visual impairment and experiences of prejudice and discrimination among adults from minority ethnic communities in the UK

**DOI:** 10.3389/fpubh.2023.1277341

**Published:** 2023-09-21

**Authors:** Nikki Heinze, Claire L. Castle

**Affiliations:** ^1^BRAVO VICTOR, London, United Kingdom; ^2^School of Music, Faculty of Arts, Humanities and Cultures, University of Leeds, Leeds, United Kingdom

**Keywords:** visual impairment, sight loss, minority ethnic, BamE, mental well-being, prejudice, discrimination, social inequalities

## Abstract

**Background:**

Visual impairment (V.I.) has been associated with a negative impact on mental health outcomes, including a process of grief among those who lose their sight. Older adults with V.I. who had experienced discrimination have been found to be at increased risk of depression, loneliness, poorer life satisfaction and poorer quality of life. Adults from minority ethnic communities (MEC) may be at increased risk of V.I. and yet, research on the experiences of MEC adults with V.I. remains limited. This article forms part of a series which explores issues and status among MEC adults living with V.I. in the UK.

**Methods:**

A secondary analysis of V.I. Lives survey data was performed to explore mental well-being assessed by the short Warwick-Edinburgh Mental Well-being scale (SWEMWBS), the emotional impact of V.I., and prejudice and discrimination among a matched control sample of 77 MEC and 77 adults from white communities (WC). Participants were matched by age, gender, UK region and urban/rural setting. Subgroup analyses were also conducted for the two largest MEC subgroups, Asian (*n* = 46) and black participants (*n* = 22).

**Results:**

There were few statistically significant differences between the groups. MEC participants were significantly more likely than WC participants to rate emotional support to come to terms with their V.I. as important and to feel optimistic about their V.I. but they were significantly less likely to agree that they were receiving the level of emotional support they needed to get on with their life. Within the MEC group, participants from Asian communities had significantly poorer mental well-being, and they were also significantly more likely to agree that the general public were often prejudiced against people with V.I. and less likely to feel optimistic about their V.I. than black participants.

**Conclusion:**

Although there were few statistically significant differences, participants from Asian communities were more likely to report poor mental and emotional well-being, and experiences of discrimination, than black and white participants. In contrast, participants from black communities fared the same as, or in some cases better than, white participants. Future research will need to confirm these findings and explore reasons for these.

## 1. Introduction

There are an estimated 2 million people living with visual impairment (V.I.) in the UK and this number is estimated to increase to approximately 4 million by 2050 (1). Adults from minority ethnic communities (MEC) have been found to be at increased risk of V.I. (2, 3) and certain eye conditions, which can lead to V.I. While prevalence of V.I. is estimated to increase in all ethnic groups, MEC adults are projected to make up an increasing proportion of adults living with V.I. in the UK (4). Yet, a rapid review of academic and grey literature published since 2005 identified substantial gaps in the evidence relating to their experiences, including experiences of prejudice and discrimination, mental well-being and the emotional impact of V.I. (5).

Across various populations, V.I. has been associated with poorer quality of life and mental health outcomes, including depression and anxiety (3, 6–12), although this may be poorer in individuals with acquired than congenital V.I. (13). Analysis of data from two large-scale UK surveys, found poorer mental well-being as assessed by the short version of the Warwick-Edinburgh Mental Well-being scale (SWEMWBS) among adults with V.I. (*M* = 22.70) compared to adults with other impairments (*M* = 24.17) and those with no impairments (*M* = 25.86) after controlling for age and sex (14). One reason for this may relate to the impact of V.I. on a wide range of areas of life including activities of daily living (15, 16), employment outcomes (3, 17), and sporting and leisure activities (18, 19). Another reason may be negative public attitudes towards people living with V.I., resulting in discrimination. Indeed, negative public attitudes arising from a lack of awareness of V.I. and its impact are perceived to be the biggest barrier to participation in everyday life by people living with V.I. (20). A UK survey of 1,223 blind and partially sighted adults found that 35% had experienced negative public attitudes in the last 12 months due to their V.I., with younger respondents and those registered as severely sight impaired or blind being more likely to report negative attitudes and unfair treatment (21). Data from the English Longitudinal Study of Ageing showed that older adults with poor eyesight who had experienced discrimination were at increased risk of depression, loneliness, poorer life satisfaction and poorer quality of life than those who had not experienced discrimination. Furthermore, older adults with poor eyesight were more likely to have experienced discrimination than those with good eyesight (22).

Whilst underexplored, MEC adults with V.I. may experience additional challenges relating to mental health, with evidence of mental health inequalities among MEC adults in the UK, both in terms of diagnoses of mental health conditions and experiences of support services (23, 24). This could reflect experiences of stigma and discrimination amongst MEC adults with V.I. arising from negative attitudes in the general population as well as those held by their own communities A small number of articles and reports provide some insights into the attitudes of individual ethnic communities of those living with V.I., and of V.I. itself (25–27), but there is no evidence relating to public attitudes and experiences of mental well-being among MEC adults in the UK. The purpose of this article is to provide preliminary insights into the emotional impact of V.I., mental well-being, and experiences of prejudice and discrimination among a sample of MEC adults living with V.I. in the UK, and to compare these to the experiences of adults from white communities (WC). It forms part of a series of articles which explore the wider experiences of MEC adults who have V.I.

## 2. Materials and methods

This article presents findings from a secondary analysis of data collected in the V.I. Lives survey. The methods of data collection and sample selection are described in more detail elsewhere (28, 29). Briefly, V.I. Lives was a UK telephone survey of people with V.I. commissioned by the Royal National Institute of the Blind (RNIB), the Thomas Pocklington Trust (TPT) and Guide Dogs for the Blind Association (Guide Dogs) and conducted by the market research agency Insight Angels and the fieldwork agency Acumen Fieldwork. Participants were recruited through the Acumen healthcare database, local and national charities, social media, and radio adverts. Non-English speakers and those without V.I. were screened out in an initial call. Data was collected in two waves, from 17 December 2019 to 23 March 2020 and from 14 August 2020 to 2 November 2020.

### 2.1. Materials

The survey explored a wide range of topics including health and well-being, functioning and accessibility, social relationships, and emotional well-being. At the end of the questionnaire participants were asked to rate the extent to which a list of issues was important to their quality of life. The list included an item on public attitudes and emotional support to come to terms with their V.I. Items were rated on a scale ranging from *extremely important* to *not important at all*.

V.I. was assessed using participants’ self-reported registration status or level of near, distance and peripheral functional vision.

Mental well-being was assessed using the short version of the Warwick-Edinburgh Mental Well-being scale (SWEMWBS) (30). SWEMWBS consists of seven positively worded items which assess thoughts and feelings, and focuses on functioning. Responses are given on a 5-point scale ranging from 1 – *None of the time* to 5 – *All of the time*. The scale is scored by summing the scores for individual items. The total raw scores are transformed into metric scores using a conversion table provided by the scale owners. Higher scores are indicative of better mental well-being. Cronbach’s alpha for this scale was 0.860, suggesting good internal consistency.

Emotional impact of V.I. was assessed by several questions. First, participants were presented with a list of 5 positive (e.g., happy and confident) and 9 negative emotions (e.g., ashamed and angry) and asked to select all that described how they felt in relation to their sight condition. Second, participants were asked to state to what extent they agreed or disagreed with a list of statements exploring their attitudes to different aspects of life on a 5-point scale ranging from *Strongly agree* to *Strongly disagree*. Five statements explored the emotional impact of V.I. (*I feel anxious about the future*, *I am struggling to come to terms with being visually impaired*, *I am able to talk to others about my vision impairment without getting upset*, *I’m often worried about what people think of me,* and *I frequently think about my vision impairment and the impact it has on my life*). One item explored the extent to which people had integrated their V.I. into their identity (*I am who I am and my V.I. is part of me*) and two assessed prejudice and discrimination (*I’ve experienced negative attitudes or discrimination due to my sight impairment* and *The general public are often prejudiced against people with sight impairment*).

### 2.2. Participants

A total of 769 participants aged 13 and over completed the survey. This secondary analysis focuses on adult participants aged 18 and over. In order to explore group differences based on ethnicity, a matched control sample was drawn using R (31). WC participants were matched to MEC participants based on their age, gender, region where they lived and whether they lived in rural areas vs. towns. One participant who did not define as male or female was excluded due to the potential for intersectionality and the lack of a match being available in the other group. The matched sample consisted of 77 MEC and 77 WC participants. The MEC group consisted of participants who identified as Asian/Asian British, Black/African/Caribbean/Black British, Mixed/multiple ethnic groups and Other ethnic group. The WC group consisted of adults who identified as White British and White other.

### 2.3. Data analysis

Data analysis was performed in IBM SPSS (32). First, descriptive statistics were produced to provide an overview of experiences within each group. Although the V.I. Lives survey was not specifically designed to explore ethnic group differences and subsample sizes for individual ethnic groups were low, subgroup analysis consisted of comparing MEC to WC participants and comparing the two MEC subgroups with the largest sample sizes, Asian (*n* = 46) and black (*n* = 22) participants. Sample sizes for all other MEC subgroups were too small to enable meaningful comparison. Response distributions were calculated as counts and proportions for each variable. Categorical variables were analysed using chi-square test. Where assumptions of cell counts were violated, Fisher’s exact test were performed in R. Ordinal variables were analysed using Mann–Whitney *U* tests. Age and the SWEMWBS score were treated as continuous variables and the mean and standard deviation for each group were calculated. Age was not normally distributed for MEC (*p* = 0.001), WC (*p* = 0.002) and Asian participants (*p* = 0.014), and the SWEMWBS scores were not normally distributed for MEC (*p* = 0.017) and WC (*p* = 0.045) participants, as assessed by Shapiro–Wilk’s test. Non-parametric Mann–Whitney *U* tests were used to assess group differences instead of t-tests. Findings relating to priority issues (29), education, employment and finances (33), health and comorbidity (34), functioning and accessibility (35), social functioning (36), and access and use of services (37) relating to this sample are presented elsewhere.

## 3. Results

Table 1 provides an overview of the characteristics of the 77 MEC, 77 WC, 46 Asian and 22 black participants. There were no statistically significant differences between MEC and WC participants, nor between Asian and black participants. Mean age was similar between WC (*M* = 41.09, SD = 15.62), MEC (*M* = 40.78, SD = 15.58), Asian (*M* = 40.17, SD = 14.61) and black (*M* = 39.18, SD = 14.70) participants. A majority across all groups were female, single, employed, residing in England, in big towns or cities, specifically in London, educated to degree level and categorised as having severe V.I. In contrast, a majority of black participants were educated to Master’s/PhD level and categorised as having moderate V.I.

**Table 1:** Participant characteristics, by subgroup.

	Asian (*n* = 46)	Black (*n* = 22)	MEC (*n* = 77)	WC (*n* = 77)
	% (*n*)	% (*n*)	% (*n*)	% (*n*)
Age	*U* = 500.5, *p* = 0.942		*U* = 2,919.5, *p* = 0.871	
*M* (SD)	40.17 (14.61)	39.18 (14.70)	40.78 (15.58)	41.09 (15.62)
Range	18–74	18–75	18–85	18–85
Gender	*Χ*^2^ (1, 68) = 0.49, *p* = 0.482		*Χ*^2^ (1, 154) = 0.00, *p* = 1.00	
Female	50.0 (23)	59.1 (13)	51.9 (40)	51.9 (40)
Male	50.0 (23)	40.9 (9)	48.1 (37)	48.1 (37)
Region	*p* = 0.789		*p* = 0.344	
London	41.3 (19)	59.1 (13)	44.2 (34)	31.2 (24)
South East	4.3 (2)	9.1 (2)	6.5 (5)	2.6 (2)
South West	6.5 (3)	–	5.2 (4)	3.9 (3)
East of England	6.5 (3)	4.5 (1)	5.2 (4)	2.6 (2)
East Midlands	2.2 (1)	4.5 (1)	3.9 (3)	5.2 (4)
West Midlands	6.5 (3)	–	5.2 (4)	2.6 (2)
North East	–	–	–	5.2 (4)
North West	17.4 (8)	9.1 (2)	13.0 (10)	23.4 (18)
Yorkshire & the Humber	4.3 (2)	4.5 (1)	3.9 (3)	3.9 (3)
Scotland	4.3 (2)	9.1 (2)	7.8 (6)	9.1 (7)
Wales	4.3 (2)	–	3.9 (3)	7.8 (6)
Northern Ireland	2.2 (1)	–	1.3 (1)	2.6 (2)
Setting	*p* = 0.234		*Χ*^2^ (2, 154) = 4.68, *p* = 0.097	
City/big town	67.4 (31)	77.3 (17)	67.5 (52)	55.8 (43)
Small town	26.1 (12)	9.1 (2)	22.1 (17)	37.7 (29)
Rural area	6.5 (3)	13.6 (3)	10.4 (8)	6.5 (5)
Education^1^	*U* = 518, *p* = 0.086		*U* = 2,794, *p* = 0.397	
No formal qualifications	–	–	–	5.2 (4)
GCSE/O-Level	15.2 (7)	4.5 (1)	11.7 (9)	14.3 (11)
A-Level /Advanced Highers	15.2 (7)	9.1 (2)	15.6 (12)	18.2 (14)
Apprenticeship, vocational, NVQ or HND	17.4 (8)	18.2 (4)	16.9 (13)	11.7 (9)
Undergraduate degree	30.4 (14)	22.7 (5)	27.3 (21)	31.2 (24)
Masters, PhD	15.2 (7)	31.8 (7)	18.2 (14)	16.9 (13)
Non-UK qualifications	4.3 (2)	–	3.9 (3)	–
Other	2.2 (1)	13.6 (3)	6.5 (5)	2.6 (2)
Employment^2^	*p* = 0.771		*Χ*^2^ (4, 154) = 0.33, *p* = 0.988	
Employed (including part-time)	41.3 (19)	54.5 (12)	42.9 (33)	40.3 (31)
Self-employed	8.7 (4)	4.5 (1)	6.5 (5)	5.2 (4)
Unemployed	19.6 (9)	9.1 (2)	14.3 (11)	14.3 (11)
Retired	6.5 (3)	9.1 (2)	10.4 (8)	11.7 (9)
Other^2^	23.9 (11)	22.7 (5)	26.0 (20)	28.6 (22)
Marital status	*p* = 0.379		*p* = 0.835	
Single	37.0 (17)	54.5 (12)	41.6 (32)	37.7 (29)
In a relationship	10.9 (5)	–	7.8 (6)	9.1 (7)
Cohabiting	8.7 (4)	4.5 (1)	6.5 (5)	10.4 (8)
Married	34.8 (16)	27.3 (6)	31.2 (24)	36.4 (28)
Civil partnership	2.2 (1)	–	2.6 (2)	–
Separated	–	4.5 (1)	1.3 (1)	1.3 (1)
Divorced	6.5 (3)	9.1 (2)	6.5 (5)	3.9 (3)
Widowed	–	–	2.6 (2)	1.3 (1)
V.I. severity^3^	*U* = 552.5, *p* = 0.516		*U* = 2,951, *p* = 0.922	
Severe	41.3 (19)	31.8 (7)	39.0 (30)	44.2 (34)
Moderate	34.8 (16)	40.9 (9)	35.1 (27)	23.4 (18)
Mild	23.9 (11)	27.3 (6)	26.0 (20)	31.2 (24)
Could not be classified	–	–	–	1.3 (1)

^1^ Statistical analysis excludes Non-UK qualifications and Other. ^2^ Due to expected frequencies of less than 5 in 5 cells (27.8%), the categories Looking after family/home, Student, Long-term sick/disabled and Unpaid work (e.g., volunteering, intern, and work experiences) were collapsed into the Other category for the statistical analysis. ^3^ Statistical analysis excludes could not be classified. MEC, minority ethnic communities (excluding white minorities), WC, White communities (including white minorities). Results for Fisher’s exact test are shown as *p*-values only.

### 3.1. Mental well-being and the emotional impact of V.I.

**Figure 1:**
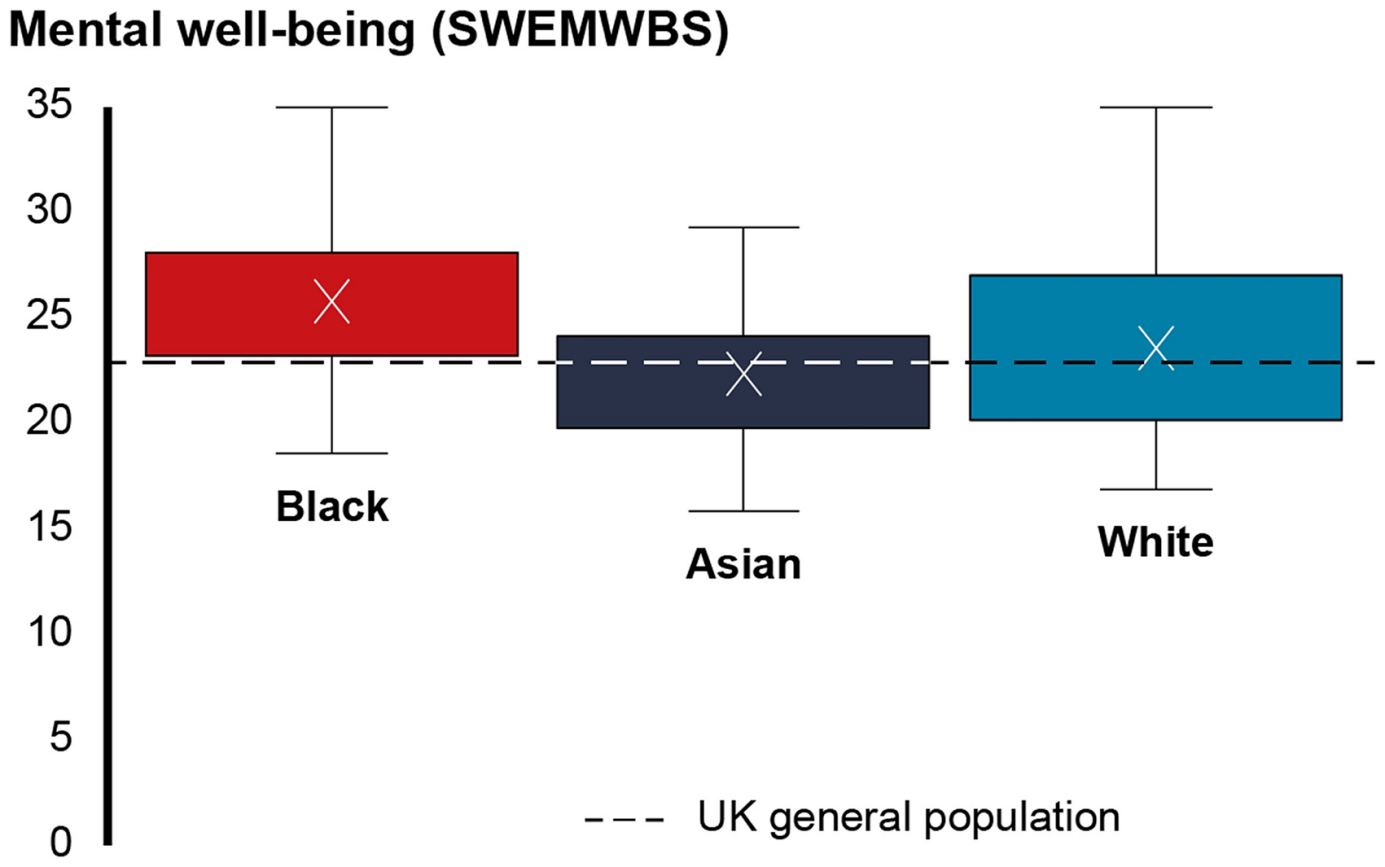
Snapshot of mental well-being by subgroup.

Table 2 shows prevalence of a range of positive and negative feelings participants had about their V.I. Table 3 gives response distributions for variables relating to the emotional impact of V.I. There was no statistically significant difference between MEC (*M* = 23.29, SD = 4.66) and WC participants (*M* = 23.58, SD = 4.81) in mental well-being as assessed by SWEMWBS, *U* = 2,773, *p* = 0.671. But within the MEC group, participants from black communities (*M* = 25.79, SD = 4.08) scored significantly higher on the SWEMWBS than participants from Asian communities (*M* = 22.37, SD = 4.21), *t*(66) = −3.170, *p* = 0.002 (Figure 1).

**Table 2:** Positive and negative feelings about V.I., by subgroup.

	Asian (*n* = 46)	Black (*n* = 22)		MEC (*n* = 77)	WC (*n* = 77)	
	% (*n*)	% (*n*)	*Χ*^2^ (1, *N* = 68)	% (*n*)	% (*n*)	*Χ*^2^ (1, *N* = 154)
Positive feelings						
Accepting	78.3 (36)	95.5 (21)	*p* = 0.089	80.5 (62)	77.9 (60)	0.16, *p* = 0.691
Determined	76.1 (35)	95.5 (21)	*p* = 0.086	79.2 (61)	84.4 (65)	0.70, *p* = 0.403
Optimistic	60.9 (28)	86.4 (19)	**4.53, *p* = 0.033**	68.8 (53)	53.2 (41)	**3.93, *p* = 0.047**
Confident	52.2 (24)	63.6 (14)	0.79, *p* = 0.373	57.1 (44)	53.2 (41)	0.24, *p* = 0.627
Happy	52.2 (24)	50.0 (11)	0.03, *p* = 0.867	51.9 (40)	53.2 (41)	0.03, *p* = 0.872
Negative feelings						
Frustrated	73.9 (34)	63.6 (14)	0.76, *p* = 0.384	68.8 (53)	76.6 (59)	1.18, *p* = 0.278
Worried	69.6 (32)	63.6 (14)	0.24, *p* = 0.625	64.9 (50)	54.5 (42)	1.73, *p* = 0.189
Sad	52.2 (24)	31.8 (7)	2.49, *p* = 0.115	45.5 (35)	40.3 (31)	0.42, *p* = 0.515
Overwhelmed	45.7 (21)	31.8 (7)	1.18, *p* = 0.278	39.0 (30)	32.5 (25)	0.71, *p* = 0.400
Angry	30.4 (14)	27.3 (6)	0.07, *p* = 0.789	28.6 (22)	32.5 (25)	0.28, *p* = 0.600
Confused	34.8 (16)	13.6 (3)	3.31, *p* = 0.069	28.6 (22)	26.0 (20)	0.13, *p* = 0.717
Rejected	32.6 (15)	13.6 (3)	2.75, *p* = 0.097	24.7 (19)	15.6 (12)	1.98, *p* = 0.159
Embarrassed	26.1 (12)	13.6 (3)	*p* = 0.353	22.1 (17)	23.4 (18)	0.04, *p* = 0.848
Ashamed	17.4 (8)	4.5 (1)	*p* = 0.253	14.3 (11)	13.0 (10)	0.06, *p* = 0.814

MEC, Minority ethnic communities (excluding white minorities); WC, white communities (including white minorities). Statistically significant results are shown in bold. Results of Fisher’s exact test are shown as *p*-values only.

**Table 3:** Emotional impact of V.I., by subgroup.

	Asian (*n* = 46)	Black (*n* = 22)	MEC (*n* = 77)	WC (*n* = 77)
	% (*n*)	% (*n*)	% (*n*)	% (*n*)
Emotional support to come to terms with V.I.	*U* = 451, *p* = 0.438		***U* = 2,446.5, *p* = 0.047**	
Extremely important	43.5 (20)	50.0 (11)	48.1 (37)	32.5 (25)
Very important	32.6 (15)	36.4 (8)	31.2 (24)	36.4 (28)
Somewhat important	15.2 (7)	9.1 (2)	13.0 (10)	20.8 (16)
Not important at all	8.7 (4)	4.5 (1)	7.8 (6)	10.4 (8)
Feels anxious about the future	*U* = 547, *p* = 0.558		*U* = 2,684, *p* = 0.356	
Strongly agree	26.1 (12)	9.1 (2)	22.1 (17)	26.3 (20)
Slightly agree	45.7 (21)	68.2 (15)	49.4 (38)	30.3 (23)
Neither/nor	13.0 (6)	–	9.1 (7)	13.2 (10)
Slightly disagree	2.2 (1)	22.7 (5)	7.8 (6)	14.5 (11)
Strongly disagree	13.0 (6)	–	11.7 (9)	15.8 (12)
Struggling to come to terms with being visually impaired	*U* = 635, *p* = 0.072		*U* = 2,946, *p* = 0.938	
Strongly agree	15.2 (7)	–	10.4 (8)	11.8 (9)
Slightly agree	21.7 (10)	9.1 (2)	19.5 (15)	19.7 (15)
Neither/nor	8.7 (4)	9.1 (2)	9.1 (7)	7.9 (6)
Slightly disagree	10.9 (5)	27.3 (6)	15.6 (12)	14.5 (11)
Strongly disagree	43.5 (20)	54.5 (12)	45.5 (35)	46.1 (35)
Able to talk to others about their V.I. without getting upset	*U* = 451.5, *p* = 0.433		*U* = 3,171.5, *p* = 0.412	
Strongly agree	50.0 (23)	63.6 (14)	53.2 (41)	53.2 (41)
Slightly agree	21.7 (10)	9.1 (2)	19.5 (15)	32.5 (25)
Neither/nor	8.7 (4)	4.5 (1)	6.5 (5)	6.5 (5)
Slightly disagree	8.7 (4)	18.2 (4)	13.0 (10)	5.2 (4)
Strongly disagree	10.9 (5)	4.5 (1)	7.8 (6)	2.6 (2)
Often worried about what people think of them	*U* = 544.5, *p* = 0.601		*U* = 3,051.5, *p* = 0.745	
Strongly agree	19.6 (9)	4.5 (1)	14.3 (11)	19.5 (15)
Slightly agree	30.4 (14)	40.9 (9)	32.5 (25)	28.6 (22)
Neither/nor	10.9 (5)	18.2 (4)	11.7 (9)	5.2 (4)
Slightly disagree	8.7 (4)	9.1 (2)	10.4 (8)	18.2 (14)
Strongly disagree	30.4 (14)	27.3 (6)	31.2 (24)	28.6 (22)
Frequently thinks about their V.I. and the impact it has on their life	*U* = 621.5, *p* = 0.109		*U* = 2,670.5, *p* = 0.270	
Strongly agree	47.8 (22)	18.2 (4)	35.1 (27)	29.9 (23)
Slightly agree	28.3 (13)	54.5 (12)	36.4 (28)	28.6 (22)
Neither/nor	2.2 (1)	4.5 (1)	5.2 (4)	10.4 (8)
Slightly disagree	8.7 (4)	13.6 (3)	9.1 (7)	18.2 (14)
Strongly disagree	13.0 (6)	9.1 (2)	14.3 (11)	13.0 (10)
I am who I am, my V.I. is part of me	*U* = 535, *p* = 0.646		*U* = 3,191, *p* = 0.231	
Strongly agree	69.6 (32)	63.6 (14)	66.2 (51)	73.7 (56)
Slightly agree	15.2 (7)	22.7 (5)	18.2 (14)	18.4 (14)
Neither/nor	8.7 (4)	–	5.2 (4)	3.9 (3)
Slightly disagree	4.3 (2)	9.1 (2)	7.8 (6)	2.6 (2)
Strongly disagree	2.2 (1)	4.5 (1)	2.6 (2)	1.3 (1)
Gets the level of emotional support needed to get on with life	*U* = 484.5, *p* = 0.770		***U* = 3,638, *p* = 0.010**	
Strongly agree	30.4 (14)	36.4 (8)	32.5 (25)	45.5 (35)
Slightly agree	32.6 (15)	31.8 (7)	29.9 (23)	35.1 (27)
Neither/nor	15.2 (7)	–	10.4 (8)	9.1 (7)
Slightly disagree	13.0 (6)	27.3 (6)	18.2 (14)	10.4 (8)
Strongly disagree	8.7 (4)	4.5 (1)	9.1 (7)	–

MEC, minority ethnic communities (excluding white minorities); WC, white communities (including white minorities). Statistically significant results are shown in bold.

**Figure 2:**
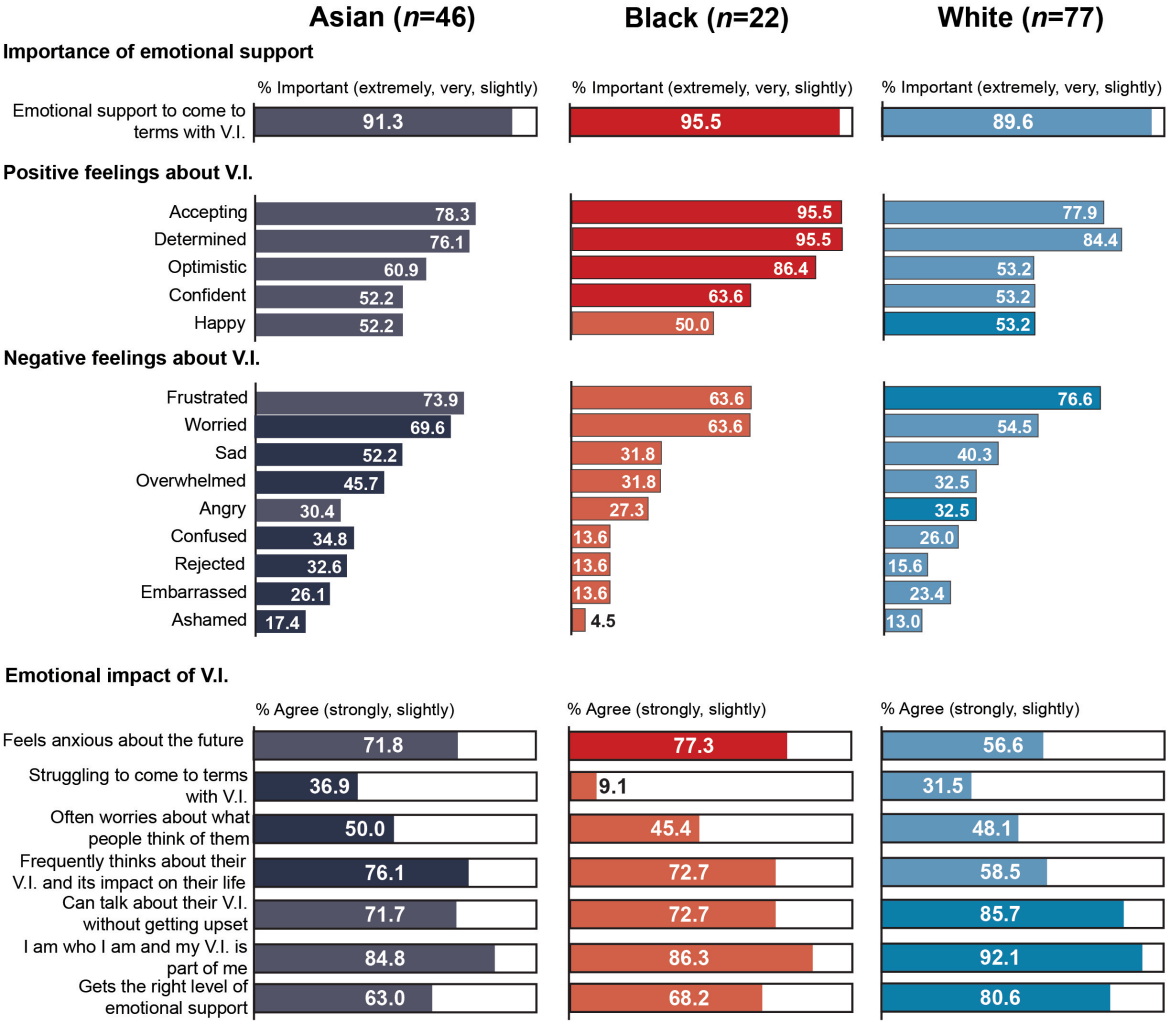
Snapshot of the emotional impact of V.I. by subgroup.

Black participants were also generally more likely to feel positive about their V.I. and less likely to feel negative about their V.I. than participants from Asian communities, although a statistically significant difference was found only for optimism, *Χ*^2^ (1, 68) = 4.53, *p* = 0.033, Cramer’s V = 0.258. As seen in Figure 2, around nine in ten black participants felt accepting, determined and optimistic about their V.I. The most common feelings among Asian participants were acceptance, determination and frustration. Despite the overall prevalence of positive feelings, over six in ten participants in both groups also felt frustrated and worried, and just over half of Asian participants also felt sad.

There were no further statistically significant differences between the two groups. Around eight in ten participants in both groups agreed that they were who they were and their V.I. was part of them. Over a third of Asian participants (36.9%) agreed that they were struggling to come to terms with being visually impaired compared to 9.1% of black participants, but this did not reach statistical significance, *U* = 635, *p* = 0.072. Moreover, Asian participants were between three and four times more likely than black participants to ‘strongly agree’ that they often worried about what people thought of them (19.6% vs. 4.5%), that they frequently thought about their V.I. and the impact it had on their life (47.8% vs. 18.2%), and that they felt anxious about the future (26.1% vs. 9.1%). Although they were slightly less likely to agree (strongly and slightly) with the last statement (71.8% vs. 77.3%) In addition, they were less likely than black participants to ‘strongly agree’ that they were able to talk to others about their V.I. without getting upset (50.0% vs. 63.6%), but the proportion who agreed (strongly and slightly) was relatively similar (71.7% vs. 72.7%).

Despite the potentially greater emotional impact of V.I. among Asian participants, emotional support to come to terms with V.I. was slightly more important to black participants, who were also slightly more likely than Asian participants to agree that they received the level of emotional support that they needed to get on with their life (68.2% vs. 63.0%). Only 4.5% of black participants, respectively, rated emotional support as ‘not important at all’ and ‘strongly disagreed’ that they got the emotional support they needed to get on with their life, compared to 8.7%, respectively, of Asian participants.

There were also few statistically significant differences when comparing MEC and WC participants. MEC participants were significantly more likely to feel optimistic about their V.I., *Χ*^2^ (1, 154) = 3.93, *p* = 0.047, Cramer’s V = 0.160, and to rate emotional support to come to terms with V.I. as important, *U* = 2446.5, *p* = 0.047. But they were significantly less likely to agree that they got the level of emotional support they needed to get on with their life, *U* = 3,638, *p* = 0.010.

Contrary to Asian and black participants, there was no clear pattern of how WC and MEC participants felt about their V.I.: WC participants were more likely than MEC participants to feel frustrated, angry and embarrassed but also determined and happy about their V.I. In contrast, MEC participants were more likely than WC participants to feel optimistic, accepting, confident, but also worried, confused, ashamed, rejected, overwhelmed and sad about their V.I. In addition, MEC participants were more likely than WC participants to agree that they felt anxious about the future (71.5% vs. 56.6%) although the latter were slightly more likely to ‘strongly agree’ (22.1% vs. 26.3%) – and to frequently think about their V.I. and its impact on their life (71.5% vs. 58.5%). MEC participants were also around twice as likely as WC participants to be unable to talk to others about their V.I. without getting upset (20.8% vs. 7.8%). But similar proportions of MEC and WC participants reported being often worried about what people thought of them (46.8% vs. 48.1%). Just under a third of participants in both groups agreed that they were struggling to come to terms with having a V.I. In relation to identity, nine in ten WC participants agreed that their V.I. was part of who they were compared to eight in ten MEC participants, who were more than 2.5 times more likely to disagree with this statement (10.4% vs. 3.9%).

### 3.2. Prejudice and discrimination

**Figure 3:**
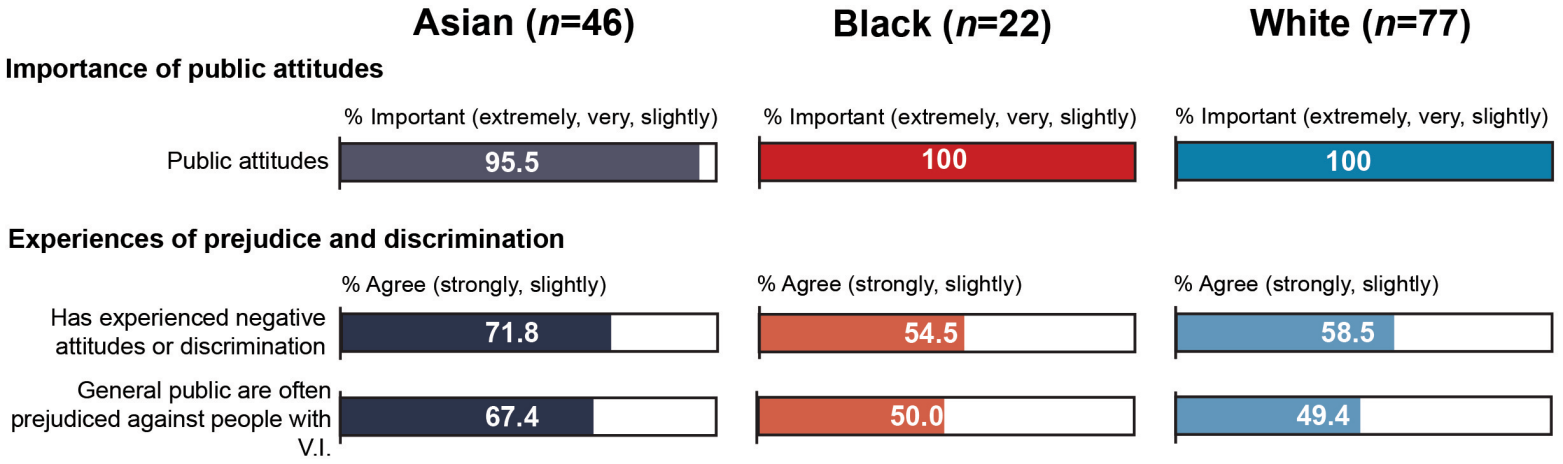
Snapshot of perceived prejudice and discrimination by subgroup.

There were no statistically significant differences between WC and MEC participants, but participants from Asian communities were significantly more likely than black participants to agree that the general public were often prejudiced against people with sight impairment, *U* = 688, *p* = 0.013 (Table 4). As seen in Figure 3, just over two thirds (67.4%) of Asian participants agreed at least slightly with this statement compared to half of black participants (50.0%). The former were just over 3.5 times more likely to ‘strongly agree’ (32.6% vs. 9.1%), and black participants were over seven times more likely to ‘strongly disagree’ (4.3% vs. 31.8%). While there were no further statistically significant differences, Asian participants were also more likely than black participants to agree that they had experienced negative attitudes or discrimination due to their sight impairment (71.8% vs. 54.5%), although participants from black communities were slightly more likely to “strongly agree” with this statement (40.9% vs. 37.0%). Similarly, Asian participants were more likely than black participants to rate understanding among the general public about how they can help people with V.I. as “extremely” or “very important” (84.4% vs. 68.2%), but none of the black participants rated this as “not important at all”, compared to 4.4% of Asian participants.

**Table 4:** Perceptions of negative attitudes and discrimination, by subgroup.

	Asian (*n* = 46)	Black (*n* = 22)	MEC (*n* = 77)	WC (*n* = 77)
	% (*n*)	% (*n*)	% (*n*)	% (*n*)
Understanding among the general public about how they can help people with V.I.	*U* = 547.5, *p* = 0.452		*U* = 2,759.5, *p* = 0.514	
Extremely important	44.4 (20)	40.9 (9)	46.1 (35)	40.3 (31)
Very important	40.0 (18)	27.3 (6)	34.2 (26)	36.4 (28)
Somewhat important	11.1 (5)	31.8 (7)	15.8 (12)	23.4 (18)
Not important at all	4.4 (2)	–	3.9 (3)	–
Has experienced negative attitudes or discrimination due to their V.I.	*U* = 566, *p* = 0.411		*U* = 2,733, *p* = 0.386	
Strongly agree	37.0 (17)	40.9 (9)	36.4 (28)	29.9 (23)
Slightly agree	34.8 (16)	13.6 (3)	27.3 (21)	28.6 (22)
Neither agree nor disagree	4.3 (2)	4.5 (1)	6.5 (5)	2.6 (2)
Slightly disagree	13.0 (6)	9.1 (2)	10.4 (8)	18.2 (14)
Strongly disagree	10.9 (5)	31.8 (7)	19.5 (15)	20.8 (16)
General public are often prejudiced against people with V.I.	***U* = 688, *p* = 0.013**		*U* = 2,688, *p* = 0.304	
Strongly agree	32.6 (15)	9.1 (2)	24.7 (19)	19.5 (15)
Slightly agree	34.8 (16)	40.9 (9)	35.1 (27)	29.9 (23)
Neither/nor	15.2 (7)	9.1 (2)	14.3 (11)	18.2 (14)
Slightly disagree	13.0 (6)	9.1 (2)	13.0 (10)	22.1 (17)
Strongly disagree	4.3 (2)	31.8 (7)	13.0 (10)	10.4 (8)

MEC, minority ethnic communities (excluding white minorities), WC, white communities (including white minorities). Statistically significant results are shown in bold.

Although there were no statistically significant differences between MEC and WC participants, experiences of negative attitudes and prejudice were slightly higher among MEC participants: 63.7% of MEC participants agreed that they had experienced negative attitudes or discrimination due to their V.I. and 59.8% agreed that the general public are often prejudiced against people with V.I., compared to 58.5 and 49.4% of WC participants, respectively. MEC participants were also slightly more likely to ‘strongly agree’ that the general public were often prejudiced (13.0% vs. 10.4%). Similar proportions of MEC and WC participants rated understanding among the general public as “extremely” or “very important” (80.3% vs. 76.7%). But 3.9% of MEC participants also rated this as “not important at all” compared to none of the WC participants.

## 4. Discussion

This article provides an overview of the emotional and mental well-being impacts of V.I. and experiences of prejudice and discrimination among a sample of MEC adults, including those from Asian and black communities, compared to WC adults. Overall, there were few statistically significant differences between the groups. MEC participants were significantly more likely than WC participants to rate emotional support to come to terms with their V.I. as important and to feel optimistic about their V.I. but they were significantly less likely to agree that they were receiving the level of emotional support they needed to get on with their life. Within the MEC group, participants from Asian communities had significantly poorer mental well-being than black participants as assessed by SWEMWBS. They were also significantly more likely to agree that the general public were often prejudiced against people with V.I. and less likely to feel optimistic about their V.I. than black participants.

The mental well-being score of MEC (*M* = 23.29, SD = 4.66) and WC participants (*M* = 23.58, SD = 4.81) was similar to the UK general population norm (*M* = 23.5, SD = 3.9) (38) and the unadjusted SWEMWBS score previously reported for people with sight loss in the UK (*M* = 23.23) (14). Encouragingly, black participants (*M* = 25.79, SD = 4.08) scored above both people with sight loss and the UK general population in terms of mental well-being. This reflects higher SWEMWBS scores among black participants relative to other ethnic groups in a general population sample (38). Compared to Asian and WC participants, black participants were most likely to feel accepting, determined, optimistic and confident and least likely to feel frustrated, sad, overwhelmed, angry, confused, rejected, embarrassed and ashamed about their V.I., although around two thirds of black participants also reported feeling frustrated and worried about their V.I. In addition, black participants were least likely to be struggling to come to terms with being visually impaired and to worry about what people thought of them. It is unclear if this reflects greater resilience, better access to support, a reluctance to report emotional and mental health difficulties and/or selection bias among this group, whereby those with better mental and emotional well-being were more willing to take part in the research. There is evidence of cultural barriers that may prevent MEC people, particularly men, from talking about emotional and mental health difficulties (39). Cross et al. (26) note the impact of intra-cultural stereotypes of Afro-Caribbean people, including women, as being proud, stoic and independent, on the sharing of family histories of V.I. and timely treatment-seeking. These stereotypes conflict with perceptions associating blindness with helplessness, social isolation, and being a victim (26). It is possible that unhelpful perceptions within and/or outside the communities may have acted as a barrier to sharing emotional difficulties in the current sample. Alternatively, black participants may benefit from better acess to support than other groups. Explorations of social functioning found that nine in ten black participants agreed that they felt supported by family and friends (36). Such informal social networks may provide a safe space to discuss emotional and mental health difficulties (39). However, almost a third of black participants disagreed that they got the level of emotional support they needed. This is considerably higher than the proportion of WC (10.4%) and also Asian participants (21.7%) who disagreed that they got the level of emotional support they needed, although the latter were more likely to disagree strongly. The importance assigned to better emotional support to come to terms with V.I. among black participants may reflect a need for more formal emotional support structures outside of the family and their social network. The extent to which V.I. impacts on the emotional and mental well-being of adults from black communities will need to be confirmed in future research, which could also explore the impact of cultural perceptions and attitudes towards health-related issues, including V.I. and mental health difficulties, on resilience and mental well-being, and the role of formal and informal support networks in meeting emotional and mental well-being needs.

In contrast, participants from Asian communities appeared to be particularly impacted by their V.I. The mental well-being score for Asian participants (*M* = 22.37, SD = 4.21) was lower than for any other group, including the wider population of people with sight loss (14) and the UK general population (38). Asian participants were more likely to feel worried, sad, overwhelmed, confused, rejected, embarrassed and ashamed, and they were less likely to feel determined and confident than both black and WC participants. This may indicate unmet emotional and mental well-being support needs among this group which could reflect several factors. Although the evidence is mixed, some studies have identified poorer mental well-being among some South Asian groups, particularly women (40, 41). For instance, Fazil and Cochrane (42) found a higher prevalence of depression as assessed by the 28 item General Health Questionnaire (GHQ-28) among a sample of Pakistani women in the West Midlands compared to white women, with levels being lower among those in employment and those who had been in living in the UK for longer. However, Asian people in England were found to be more likely to have high mental well-being as assessed by SWEMWBS relative to white people (38). While 8 in 10 Asian participants in this sample felt supported by family and friends, their support network was smaller than for the other groups and they felt significantly more isolated than black participants (36). There is evidence of stigma associated with emotional and mental health difficulties which may prevent MEC people from seeking help for these difficulties (39). Although informal social networks have been identified as a viable alternative to seeking formal mental health support (39), smaller social networks may impact on individual’s access to this informal support.

Over two thirds of Asian participants agreed that the general public were often prejudiced against people with V.I. and just under three quarters had experienced discrimination and prejudice due to their V.I. This may at least partly explain the poorer emotional and mental well-being found in this group. Negative public attitudes towards people living with V.I. have been identified as one of the biggest barriers to participation in everyday life by those living with V.I. (20), and research demonstrates the negative impact of discrimination on mental health among adults with V.I. (22). While comparatively lower, around half of black and WC participants also agreed that the public was often prejudiced against people with V.I. and over half had experienced discrimination and prejudice themselves. This is despite UK legislation to protect people with V.I. and other disabilities from discrimination. The survey does not explore what form the discrimination took, and it is not possible to draw conclusions about the impact of negative attitudes on the emotional impact of V.I. and mental well-being. But around half of participants across all groups agreed that they often worried about what people thought about them and public attitudes emerged as a priority issue among all groups. The limited available evidence (26, 27, 43) suggests that the perceptions and attitudes towards people living with V.I. held by different communities have the potential to impact on identity, adapting to sight loss, and timely treatment-seeking. For instance, treatment may be delayed among Indian communities because sight loss was viewed as being a part of the ageing process and a major disability only once there was substantial dependence and loss of function (27), while among Somali communities, V.I. was associated with stigma, related to perceptions of not existing and helplessness, which resulted in a reluctance to identify as having V.I. (25).

Unhelpful perceptions of V.I. held by the general public have also been found to impact on the extent to which people, particularly those with partial sight loss, can develop a positive V.I. identity (44). Previous research has shown that acceptance of V.I. may be associated with better well-being and lower levels of depression (45), and that a positive disability identity has been associated with a greater sense of belonging. The latter, in turn, has been associated with better physical health and mood, although these effects were mediated by self-esteem (46). In this study, most participants in all groups viewed V.I. as a part of their identity: at least eight in ten participants in all groups agreed that they were who they were and that their V.I. was part of them. However, the current data does not specify if V.I. constitutes a positive, negative or neutral part of participants’ identity.

### 4.1. Limitations

There are several sample-related limitations which impact on the generalizability of findings: findings are based on a relatively small convenience sample, which excluded non-English speakers and institutionalized adults. These individuals may be particularly vulnerable and in need of support. Subsample sizes for minority ethnic communities were particularly small, impacting on statistical power. Despite the diversity of communities included under the labels “Asian”, “black”, and “white”, it was not possible to disaggregate groups further. Findings for the Asian communities, for instance, may reflect the experiences of Pakistani but not Chinese adults.

There were additional survey-related limitations: individual survey topics (e.g., experiences of prejudice) were not explored in-depth and it is not possible to draw conclusions about the reasons for individual findings. In addition, the wording of some questions and/or response options made it difficult to interpret findings. For instance, a “slightly agree” response to the statement *I’ve experienced negative attitudes or discrimination due to my V.I.* may express uncertainty about whether a participant had experienced discrimination, relate to the frequency (occurred only once or twice), the timing (has not occurred recently), the seriousness, or the impact of the discrimination. Despite this, these questions give an indication of participants’ experiences, with the caveat that they will need to be confirmed.

### 4.2. Conclusion

Overall, there were few statistically significant differences between the groups. While participants from black communities fared the same as, or in some cases better than, white participants, participants from Asian communities were more likely to report poor mental and emotional well-being, and experiences of discrimination, than black and white participants. This highlights the need for future research with larger samples and with individual communities to confirm these findings and explore reasons for differences in mental and emotional well-being. This includes the role of public and community attitudes and perceptions of people with V.I. on well-being and identity.

## Data availability statement

The original contributions presented in the study are included in the article/supplementary material, further inquiries can be directed to the corresponding author.

## Ethics statement

Ethical approval was not required for the study involving humans in accordance with the local legislation and institutional requirements. Written informed consent to participate in this study was not required from the participants or the participants’ legal guardians/next of kin in accordance with the national legislation and the institutional requirements.

## Author contributions

NH: Conceptualization, Data curation, Formal analysis, Methodology, Visualization, Writing – original draft. CC: Visualization, Writing – review & editing.

## Funding

The author(s) declare financial support was received for the research, authorship, and/or publication of this article. This research was supported by the Thomas Pocklington Trust, grant number: FR-00380.

## Conflict of interest

The authors declare that the research was conducted in the absence of any commercial or financial relationships that could be construed as a potential conflict of interest.

## Publisher’s note

All claims expressed in this article are solely those of the authors and do not necessarily represent those of their affiliated organizations, or those of the publisher, the editors and the reviewers. Any product that may be evaluated in this article, or claim that may be made by its manufacturer, is not guaranteed or endorsed by the publisher.

## Abbreviations

UK: United Kingdom
MEC: minority ethnic communities
WC: white communities
V.I.: visual impairment
SWEMWBS: short version of the Warwick-Edinburgh Mental Well-being scale
